# High-performance room-temperature molecular switches enabled by resonant tunnelling in dithia-porphyrins

**DOI:** 10.1039/d5sc04840k

**Published:** 2025-10-17

**Authors:** Kavita Garg, Nikshay Bisht, Praveen C. Ramamurthy

**Affiliations:** a Department of Chemistry, DIT University Dehradun 248009 India kavitachemistry1@gmail.com; b Department of Materials Engineering, Indian Institute of Science Bangalore India

## Abstract

Achieving stable and reproducible single-molecule switches at room temperature remains a key challenge in molecular electronics. Conventional porphyrins, while attractive for their conjugated framework and versatile redox chemistry, often exhibit wide HOMO–LUMO gaps, weaker electrode coupling, and unstable redox states, limiting their switching performance. Here, we demonstrate that core-modified 21,23-dithia-porphyrins (N_2_S_2_-porphyrins) overcome these limitations by introducing sulfur atoms into the porphyrin core. This strategic modification lowers the HOMO–LUMO gap (better conduction), enhances orbital coupling with soft Hg electrodes, and, most importantly, stabilizes redox states that act as reliable molecular switching centers. When integrated as self-assembled monolayers on silicon, N_2_S_2_-porphyrins produce small-area MMS junctions that exhibit room-temperature bistable I–V behaviour with ON/OFF ratios >20, narrow SET thresholds (∼0.6 V), and stability over 1000 cycles. Supported by DFT–NEGF calculations, these results establish core-modified dithia-porphyrins as superior to conventional porphyrins for molecular-scale memory and logic devices. This study positions core-modified dithia-porphyrins as a new molecular design paradigm, where stable redox chemistry and improved device reproducibility converge to realize practical room-temperature molecular electronics.

## Abstract 2

Core-modified N_2_S_2_-porphyrins enable stable, high-performance molecular switches with sharp SET thresholds, ON/OFF > 20, and durable cycling at room temperature.

## Graphical abstract

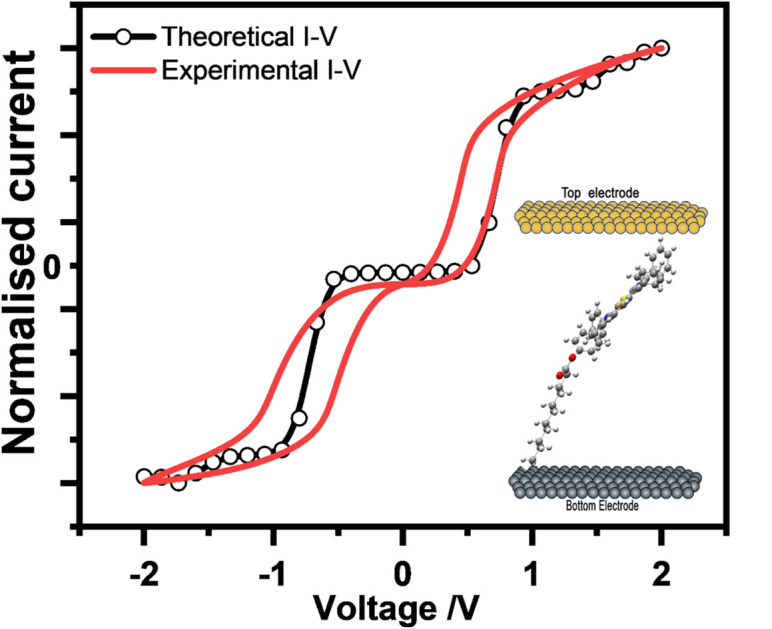

## Introduction

Molecular electronics aspires to harness individual molecules as functional circuit elements, pushing electronic devices to the ultimate limit of miniaturization. Among its most compelling components are molecular switches—molecules capable of reversibly toggling between discrete conductive states in response to external stimuli such as electric fields, light, or chemical inputs.^1–6^ Such switches underpin the vision of molecular-scale memory and logic. Yet, despite substantial progress, most conductance-based switches remain constrained by low ON/OFF ratios, poor reproducibility, unstable redox states, and a reliance on cryogenic conditions to observe quantum-level transport phenomena.^7–9^ Overcoming these limitations requires molecular frameworks that unite intrinsic redox bistability with strong and stable electrode coupling, while maintaining operational robustness under ambient conditions.

Porphyrins represent an attractive molecular scaffold owing to their rigid planar geometry, extended π-conjugation, and versatile redox chemistry.^10–13^ However, conventional tetrapyrrolic porphyrins often suffer from wide HOMO–LUMO gaps, weak orbital overlap with soft electrodes, and unstable redox behaviour, leading to modest and irreproducible switching. To address these shortcomings, we introduce a core heteroatom engineering strategy, in which two pyrrolic nitrogens are replaced with sulfur atoms, yielding 21,23-dithia-porphyrins (N_2_S_2_-porphyrins). This subtle yet powerful modification lowers the HOMO–LUMO gap, stabilizes multiple redox states, and significantly enhances electronic coupling to soft electrodes (*e.g.*, Hg, Au) due to sulfur's superior anchoring ability.^14,15^ The resulting macrocycle is transformed into a redox-active framework intrinsically suited for reproducible, room-temperature molecular switching.

Most molecular switches are studied at the single-molecule level using scanning tunnelling microscopy (STM). However, such isolated switches often suffer from limited thermal stability and low lateral diffusion barriers, posing challenges for their integration into scalable devices.^6,16^ To achieve high data storage density, a molecular switch must exhibit intrinsic bistability and concurrently possess the capability to form a robust and ordered self-assembled monolayer (SAM).^17^ Recent advancements have highlighted that intermolecular interactions—particularly halogen and hydrogen bonding—can significantly enhance the structural stability of molecular assemblies while retaining the responsiveness required for single-molecule manipulation.^18^ A well-designed self-assembled molecular switch can therefore offer a combination of high thermal stability, increased storage density, and improved operational control.^19^ To implement such molecular architectures in practical device platforms, bottom-up fabrication approaches utilizing SAMs are increasingly employed. While conventional metal–molecule–metal (MMM) junctions have been extensively studied, there is growing interest in metal–molecule–semiconductor (MMS) junctions due to their fabrication simplicity and seamless integration with silicon-based electronics.^20–23^

Covalent anchoring of molecular layers to oxide-free silicon surfaces offers enhanced electronic coupling and mitigates interfacial charging artefacts. A variety of surface functionalization techniques have been developed to enable covalent linkage of organic molecules to silicon substrates, including thermally or photochemically induced hydro-silylation of alkenes/alkynes with Si–H bonds, radical-initiated processes, electrochemical methods,^24–27^ and Grignard reactions.^28^ Among these, electrochemical grafting has emerged as a particularly attractive strategy due to its precise control over reaction parameters and its inherent ability to suppress surface oxidation during SAM formation.^29^

In this study, we fabricate self-assembled monolayer (SAM)-based metal–molecule–semiconductor (MMS) junctions by electrochemically grafting a tailor-made dithia-tetraphenyl-porphyrin (N_2_S_2_-TPP) derivative onto hydrogen-terminated silicon surfaces *via* an alkene-functionalized carbon linker. A soft mercury (Hg) top contact is employed to complete the junction, enabling the characterization of charge transport properties. Strategic replacement of two inner nitrogens of the porphyrin core with sulfur atoms yields the N_2_S_2_-TPP framework, which features a narrowed HOMO–LUMO gap, more stable redox states, and stronger coupling with soft electrodes, properties that are not attainable with conventional porphyrins. These junctions exhibit reproducible bistable I–V behaviour with sharp conductance transitions and high ON/OFF ratios at room temperature. Our results demonstrate that core heteroatom engineering is a simple yet powerful strategy, establishing dithia-porphyrins as a superior molecular platform for stable, room-temperature molecular switches and next-generation electronic devices.

## Results and discussions

### Synthesis

A meso-(*para*-hydroxyphenyl) substitution of dithia-porphyrin was chosen to provide a synthetically accessible anchoring site while leaving the dithia-porphyrin core electronically intact. Meso substitution preserves macrocycle planarity and redox properties, while the *para*-hydroxy group enables clean derivatization to a C_11_ alkenyl ester for Si grafting. The C_11_ linker length offers an optimal balance, long enough to form dense, defect-minimized SAMs and suppress interfacial traps, yet short enough to maintain efficient tunnelling and sharp switching thresholds.^29–31^ The remaining meso-phenyl substituents enhance solubility and reduce uncontrolled aggregation, ensuring ordered monolayer formation. This design thus secures reliable anchoring, stable redox activity, and efficient charge transport through the N_2_S_2_ core.

**Scheme 1:**
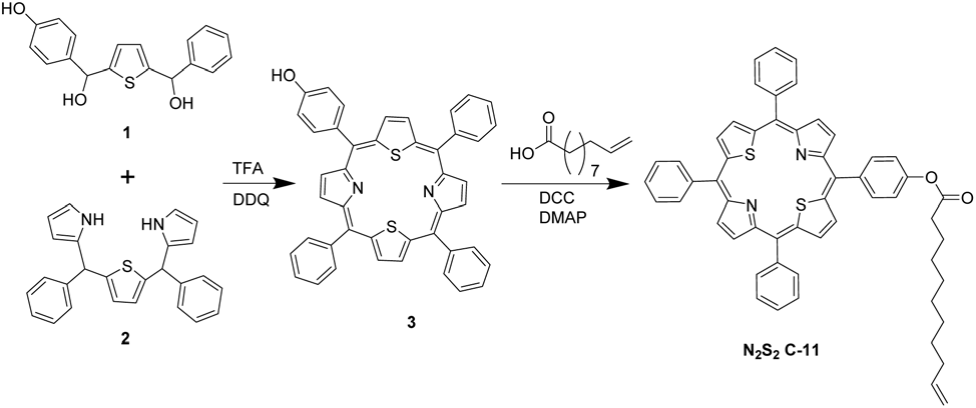
Synthetic Scheme of 5,10,15,20-tetraphenyl-21,23-dithia-porphyrinyl undec-10-en-oate (N_2_S_2_ C-11); Step one shows condensation of thiophene diol 1 (*p*-hydroxyphenyl/phenyl) with diphenyl-16-thiatripyrrane 2 under Adler's and step 2 shows esterification with 10-undecenoic acid.

The desired N_2_S_2_–C_11_ porphyrin was synthesized from a mono-substituted porphyrin obtained by condensing unsymmetrical thiophene diol 1 (*p*-hydroxyphenyl/phenyl) with diphenyl-16-thiatripyrrane 2 under Lindsey's^32^ or Adler's porphyrin^33^ conditions (Scheme 1). TLC of the crude product showed a distinct spot for the target mono-hydroxyphenyl–triphenyl-21,23-dithia-porphyrin alongside polymeric by-products. After work-up and silica gel chromatography, 5-(4-hydroxyphenyl)-10,15,20-triphenyl-21,23-dithia-porphyrin (3) was isolated in 8–12% yield. Esterification of 3 with 10-undecenoic acid under DCC/DMAP coupling gave the final N_2_S_2_–C_11_ porphyrin for SAM fabrication.

### Fabrication of device

**Fig. 1:**
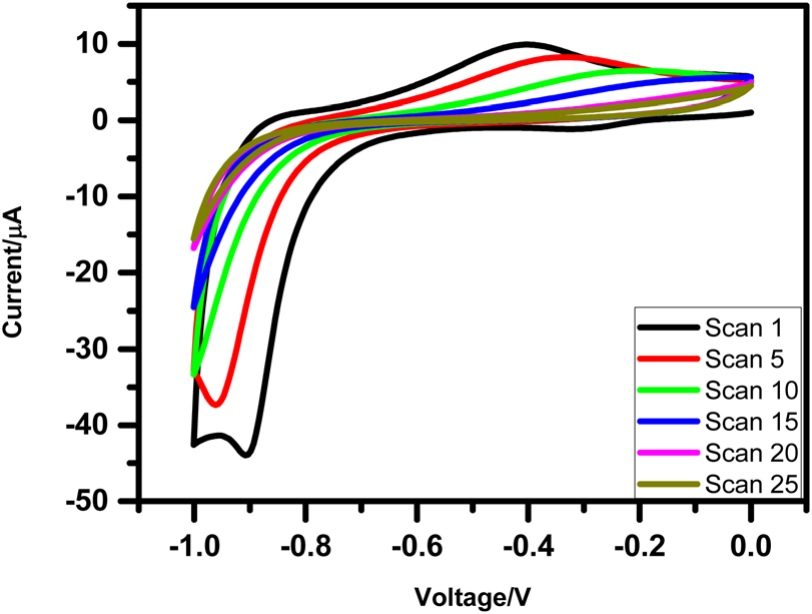
CVs indicating electro-grafting of N_2_S_2_-TPP-C-11 molecule on silicon (*n*^++^) wafer. The deposition was carried out by CV at a scan rate of 0.05 V s^−1^ under N_2_ atmosphere using Si wafers as the WE, Pt as the CE, and Ag/AgCl as the RE, and 0.1 M Bu_4_NP as the electrolyte, using the N_2_S_2_ (1 mM) in dry CH_2_Cl_2_.

After synthesis, N_2_S_2_–TPP–C_11_ was electro-grafted onto H-terminated silicon using established protocols for alkene/aryl electro-grafting on silicon surfaces,^29,34^ where a three-electrode configuration with the Si–H wafer as the working electrode (WE), Pt as the counter electrode (CE), and Ag/AgCl as the reference electrode (RE). The grafting was performed in dry CH_2_Cl_2_ containing 1 mM N_2_S_2_–porphyrin derivative and 0.1 M tert-butylammonium perchlorate as the supporting electrolyte. (Mechanism shown in the SI (Fig. S6)). Application of a negative bias (0 to −0.1 V) favours the formation of stable Si–C bonds, while the reducing potential simultaneously suppresses Si oxidation, thereby ensuring oxide-free deposition. The progress of deposition was monitored by cyclic voltammetry (Fig. 1), where the gradual disappearance of the oxidation peak at −0.3 V confirmed completion of monolayer formation. Control experiments with 1-undecene exhibited a similar oxidation feature, whereas no such response was observed in the blank electrolyte. Atomic force microscopy (AFM) confirmed the formation of a compact, homogeneous monolayer after ∼25 scans, while extended cycling led to multilayer growth, as also reported for similar electro-grafted systems.^30,35,36^ Following deposition, the electrodes were sequentially sonicated in CH_2_Cl_2_ and isopropanol (10 min each) to remove electrolyte residues and physisorbed porphyrin molecules.

### Characterization of the monolayers

**Fig. 2:**
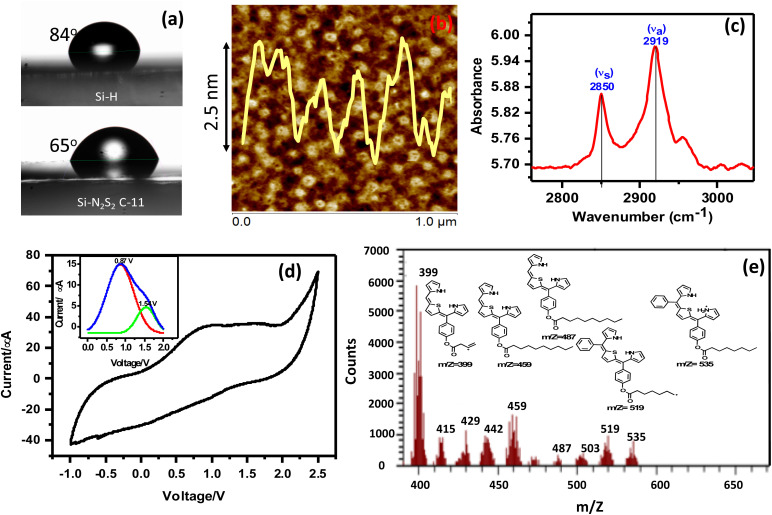
Characterization of N_2_S_2_–C_11_ monolayers on Si wafers. (a) Contact angle measurements of H-terminated silicon (Si–H) and N_2_S_2_–C_11_ monolayer-grafted Si wafers, showing a decrease from 84° to 65°, indicative of increased surface hydrophilicity upon functionalization. (b) AFM topography images (1 μm × 1 μm) of N_2_S_2_–TPP–C_11_ monolayers on Si(111); the line profile overlay was used to estimate the monolayer thickness from the depth of pinholes. (c) Polarized FTIR spectra of N_2_S_2_–C_11_ monolayer-grafted Si wafer, showing CH_2_ symmetric and asymmetric stretching bands at 2850 and 2915 cm^−1^, consistent with rigid, solid-like alkyl chain packing and providing insights into molecular orientation. (d) Fast-scan cyclic voltammograms (10 V s^−1^) of N_2_S_2_–TPP–C_11_ monolayers electro-grafted on *n*^++^ silicon wafers, recorded under N_2_ using Pt as the counter electrode (CE), Ag/AgCl as the reference electrode (RE), and 0.1 M Bu_4_NP as the electrolyte; insets show magnified redox peaks after background correction. (e) SIMS of the monolayers of N_2_S_2_-TPP-C-_11_ electrografted on a silicon (*n*^++^) wafer.

To confirm the successful formation of N_2_S_2_–TPP–C_11_ monolayers on Si, the electro-grafted surfaces were characterized using multiple complementary techniques, including contact angle measurements, AFM, ellipsometry, polarized FTIR, XPS/UPS, SIMS, and electrochemistry. Contact angle measurements revealed a significant decrease from 84° for clean Si to 65 ± 2° for the N_2_S_2_–TPP–C_11_-grafted surface, indicating increased hydrophilicity (Fig. 2(a)). In contrast, the C_11_ alkyl monolayer exhibited a higher contact angle of 112°.^37–39^ The lower contact angle for N_2_S_2_–TPP–C_11_ suggests that the molecules are slightly tilted on the surface, exposing the pyrrole and thiophene rings, which can interact with the surrounding environment or top contacts. These values are consistent with previously reported thiophene-terminated alkyl monolayers prepared *via* late-stage aryl attachment (66–74°), confirming the chemical integrity and orientation of the grafted molecules.^39,40^

AFM analysis revealed that the N_2_S_2_–TPP–C_11_ monolayers, formed after 25 scans, are well-organized with large grain sizes and small pinholes (>100 nm^2^). The monolayer thickness, determined from the depth of these pinholes, was ∼2.5 ± 0.3 nm (Fig. 2(b)), in excellent agreement with ellipsometry measurements (2.5 ± 0.2 nm). This value is slightly below the theoretical molecular length (∼2.8 nm) obtained from gas-phase DFT optimization, indicating that the porphyrins adopt a near-vertical orientation with a small tilt angle of ∼15° from the surface normal. Such orientation ensures partial exposure of pyrrole and thiophene moieties to the electrode and facilitates strong π–π interactions between neighbouring porphyrins, underpinning the monolayer's structural integrity.

Polarized FTIR spectra provide further insight into the alkyl chain ordering. In solid-like alkyl monolayers, CH_2_ stretching modes typically appear at 2848–2852 cm^−1^ (*ν*_s_) and 2916–2920 cm^−1^ (*ν*_a_) whereas liquid-like, disordered chains shift to higher wavenumbers (∼2853–2855 cm^−1^ and 2922–2925 cm^−1^) due to out-of-plane C–C bond twisting. The polarized FTIR of N_2_S_2_–TPP–C_11_ monolayers (Fig. 2(c)) shows *ν*_s_ = 2850 cm^−1^ and *ν*_a_ = 2919 cm^−1^, confirming rigid, well-packed, solid-like alkyl chains. The longer C_11_ chains promote this ordered packing, further stabilizing π–π interactions between porphyrin cores.

The molecular density of N_2_S_2_–TPP–C_11_ monolayers was determined using fast-scan cyclic voltammetry (CV, 10 V s^−1^, Fig. 2(d)), showing two irreversible oxidation peaks at +0.8 V and +1.5 V, consistent with solution-phase behaviour (Fig. S7), while blank Si and C_11_ alkyl monolayers showed no redox activity. Integration of the CV peaks and normalization by the scan rate yielded a surface coverage of 5.9 × 10^13^ molecules cm^−2^, corresponding to an area per molecule of ∼160 Å^2^. This is significantly larger than the ∼24 Å^2^ per molecule reported for simple C_12_–C_18_ alkyl monolayers on Si(100), but closely matches the reported area for face-on porphyrin packing (∼160 Å^2^), over edge-on orientation (∼70 Å^2^ per molecule).^41^ These results confirm that the porphyrins adopt a densely packed, face-on configuration.

XPS analysis further confirmed monolayer formation, showing covalent Si–C bonds at 100.5 eV and no SiO_2_ signal (103 eV), with C, N, O, and S signals consistent with the expected monolayer composition (Fig. S8).^25,42^ UPS measurements indicate a HOMO level of −5.21 eV (Fig. S9), in excellent agreement with the value obtained from CV and with reported values for dithia-porphyrins.^15^ Together, these results demonstrate that the porphyrin electronic structure is preserved and remains well decoupled from the silicon substrate, likely due to C_11_ alkyl chains acting as spacers that prevent strong electronic coupling with the silicon substrate.

The SIMS showed peaks at *m*/*z* 535, 519, 487, 459, and 399 amu corresponding to different fragments of the N_2_S_2_–TPP–C_11_ molecule (Fig. 2(e)) (detailed SIMS 200–1000 amu and fragmentation given in supplementary (Fig. S10)). The *m*/*z* fragmentation analysis shows that the N_2_S_2_ porphyrin ring is fragmented while the relatively long alkyl chains remain intact, in contrast to other porphyrin-based monolayers where intact porphyrin and shorter fragments suggest tilted orientations.^30,31,36^ This pattern indicates that the present monolayers are densely packed with molecules standing upright at a low tilt angle, which is consistent with AFM, Ellipsometry, and CV data.

### *I*–*V* measurement

**Fig. 3:**
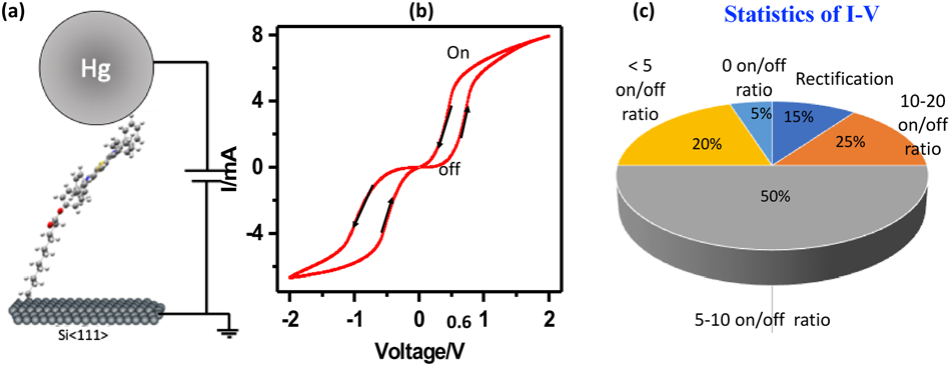
(a) *I*–*V* measurement set up for the device Si(*n*^+^)/N_2_S_2_ C11/Hg, where molecule has tilt angle of ∼15°. (b) Experimental *I*–*V* measurement in voltage sweep (0 → 2 V, 2 V→ −2 V, −2 V → 0 V), to check if features in the *I*–*V* behaviour originate from the polarity of the initial voltage that is applied and from the scan direction. (c) Statistics of 100 devices, each junction was scanned 100 scans to check the stability of the devices.

To investigate the charge transport properties and switching potential of the dithia-porphyrin (N_2_S_2_) molecule, small-area molecular junctions were fabricated. As depicted in Fig. 3(a), the device consists of a self-assembled monolayer (SAM) of N_2_S_2_-TPP-C-11 terminated molecules covalently grafted onto an (*n*^++^)-doped silicon substrate, with a liquid mercury (Hg) droplet serving as a soft, non-destructive top contact. This metal/N_2_S_2_–TPP-C_11_/Si(*n*^++^) configuration, referred to as the MMS (metal–molecule–semiconductor) architecture, enables the measurement of the collective electrical behaviour of a small ensemble of molecules. Area of mercury drop touching the surface of the grafted monolayer measured as 0.03 ± 0.005 mm^2^ through a built-in microscope, which is much higher than the pinholes (∼100 nm^2^) measured by AFM. Since the Hg drop cannot deform in such a small area, due to high surface tension, penetration through the pinholes of the SAMs is unlikely.^43^ Thus, the measured current–voltage (*I*–*V*) characteristics are attributed to direct charge transport through the molecular layer.

The current–voltage (*I*–*V*) characteristics of the N_2_S_2_–TPP molecular junction, measured across a voltage sweep sequence (0 → +2 V → −2 V → 0 V; Fig. 3(b)), device behaviour was checked by reversing the polarity of the initial voltage that is applied and from the scan direction. Device exhibits a clear and reproducible hysteresis loop for >1000 scans (Fig. S8), indicative of bistable molecular switching and non-volatile memory functionality. In forward bias, the device remains in a low-conductance (OFF state) until a threshold voltage (*V*_set_ ≈ +0.6 ± 0.05 V) triggers a sudden ∼20-fold increase in current, signalling the transition to a high-conductance (ON state). As the bias returns toward zero, the ON state persists, and upon sweeping negatively (0 → −2 V), a symmetric RESET transition occurs at ∼−0.6 ± 0.05 V, reverting the junction to the OFF state. This bidirectional switching implies that the HOMO alignment with the electrode Fermi levels is field-driven in both bias polarities.

Despite the asymmetry in electrode composition and coupling—namely, a covalent Si–C linkage through the C_11_ spacer at the bottom electrode and a noncovalent soft–soft Hg–S interaction at the top—the redox-active N_2_S_2_–TPP core remains comparably coupled to both contacts. This effective dual coupling enables reversible HOMO-mediated transport under both positive and negative bias, resulting in the relatively symmetric *I*–*V* response observed.

The device achieves an ON/OFF ratio of approximately 20, and the enclosed hysteresis confirms its memristive behaviour. Among 100 devices tested (Fig. 3(c)), ∼90% exhibit hysteresis: ∼15% of devices show ON/OFF ratios in the range of 10–20, ∼50% fall between 5–10, ∼20% are below 5, and ∼5% show no switching. Notably, the switching voltage (*V*_set_) clusters tightly around ±(0.6 ± 0.05) V for memristive devices, indicating exceptional reproducibility.

Since experimental validation of the underlying mechanism in such nano-devices through *in situ* spectroscopic measurements of redox states remains technically formidable and is currently a key limitation, this highlights an important direction for future investigations. In the present study, we have therefore employed theoretical simulations to substantiate and validate the proposed mechanism.

**Fig. 4:**
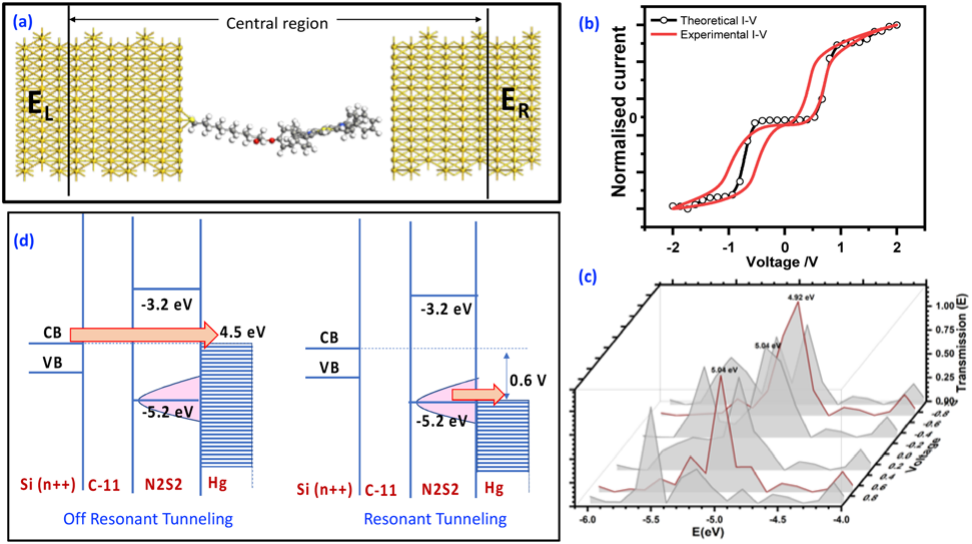
(a) Theoretical *I*–*V* measurement setup for the device Au/N2S2 C-11/Au. Alignment of the porphyrin molecule is kept to face on, and DFT optimised the DFT-optimized geometry was used for NEGF *I*–*V* calculation. (b) Theoretical *I*–*V*, *I*–*V* was calculated from −2 to 2 V with equally spaced 31 points (C). The transmission spectrum was calculated at all the 31 voltages equally spaced between −a 2 to 2 V. Data at a step size of 0.4 V is presented for clarity. (d) Representation of an energy level diagram to show the mechanism. It shows that below 0.6 V energy levels are not aligned, so we observe tunnelling of electrons. After ∼0.6,V when the HOMO level of the molecule starts aligning with the Fermi level of the Hg electrode, resonance tunnelling is observed.

Charge transport calculations were performed using ATK Toolkit, a model Au/molecule/Au junction (Fig. 4(a)), allowing determination of the transmission spectra (Fig. 4(d)) and theoretical *I*–*V* characteristics of N_2_S_2_–TPP–C_11_ (Fig. 4(c)). The ground-state geometry of the molecule was first optimized by *ab initio* molecular orbital theory and density functional theory (DFT) using Gaussian. Geometry optimization of N_2_S_2_–TPP–C_11_ was carried out without any symmetry constraints at the B3LYP/6-31G(d,p) level of theory (Fig. S12). The calculated HOMO energy is in good agreement with the values obtained experimentally from ultraviolet photoelectron spectroscopy (UPS) and cyclic voltammetry (CV) in the solid state, consistent with previous reports on porphyrin derivatives.^15,29,31^ However, the HOMO–LUMO gap predicted by DFT is noticeably larger than the experimental value. This discrepancy is a well-known artefact of DFT, arising from the neglect of electronic polarization and screening effects present in the condensed phase, which effectively reduce the transport gap. Secondly, the transport characteristics were computed by constructing a device model using the optimized molecular configuration that constitutes the central region between two electrodes. Besides the active parts of the device, the central region also includes sufficient parts of the contacts, such that the properties of the electrode regions can be described as bulk materials. This can be ensured by extending the central region into a few layers of the metallic contacts.

The calculation of the electron-transport properties of the system is divided into two parts: (i) a self-consistent calculation for the electrodes with periodic boundary conditions in the transport direction, and (ii) a self-consistent open boundary calculation of the properties of the central region, where the electrodes define the boundary conditions. The complete details of the method are described in the literature.^44^

In the actual experimental setup, the bottom contact is a heavily doped Si substrate, which may undergo surface reconstruction, while Hg, being a liquid metal, presents a complex interfacial structure. These features present significant challenges for accurate modelling using *ab initio* methods. Therefore, gold (Au) was chosen for the theoretical model due to its stable face-centered cubic structure and well-established pseudopotentials, which have been validated extensively for molecular electronics applications. Although the Au-based model does not exactly replicate the Si/Hg experimental system, it provides qualitative insights into the charge transport behaviour. Notably, Zheng *et al.*^45^ demonstrated that similar negative differential resistance (NDR) behaviour in C_60_-based molecular devices is largely independent of the electrode material; similar results have been observed by Cahen *et al.* and us,^46–48^ supporting the relevance of the current model.

In the computational model, a thiol-terminated N_2_S_2_–C_11_ molecule was used to ensure covalent bonding with the Au electrodes. The interface geometry between the thiol group and the Au surface was optimized for optimal orbital overlap. Previous studies on methyl thiol adsorbed on Au (111) surfaces^49^ indicated an optimal S–Au distance of 2.52 Å at the hollow site. The device was modelled using two Au (111) −(5 × 5) slabs with periodic boundary conditions representing the left and right electrodes. Each electrode consisted of three atomic layers, while the central scattering region included the molecule and two additional gold layers from each electrode. Electron transport properties were computed using the ATK 11.2.3 software package, employing a semi-empirical extended Hückel method combined with non-equilibrium Green's function (NEGF) formalism.^31,44^

A *k*-point mesh of 100 was used in the transport (*Z*) direction. The theoretical *I*–*V* characteristics closely resemble the experimental curve and is highly symmetrical, as shown in the overlay (Fig. 4(b)), indicating that the switching behaviour is inherent to the molecular structure. Slight differences in the voltage thresholds and slight deviation in *I*–*V* are likely due to disparities in electrode composition (Si/Hg in experiment *vs.* Au in theory). Thus, it is the electrode–molecule coupling and energetic alignment of the frontier orbitals that primarily dictate charge transport, while the alkyl spacer serves only to spatially decouple the porphyrin core from the substrate rather than influencing conduction through its dielectric character.^31,46,47^

The hysteresis observed in the experimental *I*–*V* traces is attributed to conformational changes on the oxidation of the molecule. DFT-based studies on heteroatom-substituted porphyrins, including dithia-porphyrins, have shown that upon oxidation, the macrocycle undergoes non-planar distortions such as saddling or ruffling, accompanied by a 10–20° shift in the dihedral angle between the N_2_S_2_ arm and the core.^50,51^ These structural rearrangements modulate electronic coupling with the electrodes by altering both through-bond and through-space pathways, thereby influencing charge transport. Such oxidation-induced conformational flexibility provides a molecular basis for the observed hysteresis and bistable switching, where redox-driven structural reorganization stabilizes distinct conductance states. The slight asymmetry between the forward and reverse sweeps arises due to the different work functions and interfacial properties of the top (Hg) and bottom (Si) electrodes.

Analysis of the transmission spectrum (Fig. 4(d) and S11) reveals that a molecular orbital at ∼5.2 eV exhibits the most significant change during the voltage sweep, suggesting that charge transport around 0.6 V predominantly occurs through this level. This orbital corresponds to the HOMO, as independently confirmed by DFT in the gas phase (−5.18 V), cyclic voltammetry (CV, ∼−5.25 eV), and ultraviolet photoelectron spectroscopy (UPS, −5.21 eV).^52^ This correspondence of HOMO value obtained from DFT is consistent with literature reports.^15,29,31^ The N_2_S_2_–C_11_ molecule exhibits a HOMO–LUMO gap of ∼2 eV (estimated from UV, DFT band gap is 2.6 eV) and conduction is *via* tunnelling. At *V*_set_, HOMO of the molecule aligns with the Fermi level of electrodes (Fig. S12) induces oxidation of the electron-rich N_2_S_2_–TPP core, forming a stable cation (N_2_S_2_–C_11_^+^), marked as the ON state. The N_2_S_2_–C_11_^+^ state remains, allowing the molecule to retain its conductive ON state. The long alkyl spacer electronically decouples the N_2_S_2_–TPP unit from the substrate, suppressing charge recombination and enhancing memory retention. On applying reverse voltage, it neutralizes the cation, returning the molecule to its initial OFF state (Fig. 4(b)).

## Conclusions

In conclusion, this work establishes core-modified 21,23-dithia-porphyrins (N_2_S_2_–TPPs) as a powerful molecular design strategy to achieve stable and reproducible bistable switching at room temperature. Replacing two central nitrogen's with sulphur atoms reorganizes the frontier orbitals, lowers the HOMO–LUMO gap, enhances coupling with soft Hg electrodes, and stabilizes multiple redox states that act as reliable switching centres. Integrated as covalently anchored SAMs on silicon, N_2_S_2_–TPPs deliver robust MMS junctions that exhibit reproducible bistable I–V behaviour with ON/OFF ratios >20, narrow SET thresholds (∼0.6 V), endurance beyond 1000 cycles, and >90% device yield across 100 junctions. DFT and NEGF calculations confirm that the observed switching arises from HOMO alignment of the redox-active dithia-porphyrin core with the electrode Fermi levels, validating the molecular origin of the bistability.

When compared with prior approaches, these devices demonstrate a unique balance of performance and practicality. STM and EC-STM based single-molecule switches have provided valuable mechanistic insight but typically show ON/OFF ratios in the range of 2–10 with strong tip dependence, poor reproducibility, and no scalability.^53–55^ Ionic-liquid-gated molecular switches have achieved much higher ON/OFF ratios (>100), but their reliance on liquid gating and nanogap electrodes restricts practical integration.^56^ In contrast, the present solid-state SAM-based devices combine moderate but reproducible ON/OFF ratios (>20) with exceptional device robustness, reproducibility, and scalability, thereby bridging the gap between fundamental molecular-scale studies and technologically relevant device architectures.

Together, these findings establish core heteroatom modification of porphyrins as a generalizable molecular design principle, where orbital engineering and optimized electrode coupling converge to deliver robust, room-temperature solid-state molecular switches. This work thus marks an important step toward the practical realization of molecular-scale memory and logic devices.

## Author contributions

Kavita Garg conceived and designed the study, developed the methodology, performed the investigation, curated and analysed the data, and prepared the original manuscript. Nikshay Bisht contributed to data analysis and manuscript preparation. Praveen C. Ramamurthy acquired project funding, provided laboratory infrastructure, and contributed to manuscript writing. All authors contributed to reviewing and editing the manuscript and approved the final version for publication.

## Conflicts of interest

There are no conflicts to declare.

## Supplementary Material

SC-OLF-D5SC04840K-s001

## Data Availability

The data supporting this article have been included as part of the supplementary information (SI). Supplementary information: experimental details, characterisation data (NMR, ESI-MS, SIMS, XPS, UPS, CV, I-Vs, electron density maps, and transmission spectrum). See DOI: https://doi.org/10.1039/d5sc04840k.
