# Supplementary material for: High-performance room-temperature molecular switches enabled by resonant tunnelling in dithia-porphyrins
Source: Chem Sci. 2025 Oct 17;16(47):22490–7. doi: 10.1039/d5sc04840k (PMC12551673; doi:10.1039/d5sc04840k)
Supplement: SC-016-D5SC04840K-s001 [file SC-016-D5SC04840K-s001.pdf]

Supplementary Information (SI) for Chemical Science.
This journal is © The Royal Society of Chemistry 2025

Supplementary Material
Highly stable Molecular Memories based on 21,23-Dithia-porphyrins

S. No	Content
1	**Figure S1.** ^{1}H NMR of 5-(4-hydroxy phenyl)10,15,20-triphenyl-21,23-dithiaporphyrin
2	**Figure S2.** ^{1}H NMR of 5-(4-phenyl)10,15,20-triphenyl-21,23-dithiaporphyrinyl undec10-enanoate (N_2S_2C-11).
3	**Figure S3.** ^{13}C NMR of 5-(4-phenyl)10,15,20-triphenyl-21,23-dithiaporphyrinyl undec10-enanoate (N_2S_2C-11).
4	**Figure S4.** ESI-MS of 5-(4-hydroxy phenyl)10,15,20-triphenyl-21,23-dithiaporphyrin
5	**Figure S5.** ESI-MS of 5-(4-phenyl)10,15,20-triphenyl-21,23-dithiaporphyrinyl undec10-enanoate (N_2S_2C-11)
6	**Figure. S6.** Mechanism of monolayer formation
7	**Figure. S7.** CV of N2S2 solution
8	**Figure S8.** XPS of 5-(4-phenyl)10,15,20-triphenyl-21,23-dithiaporphyrinyl undec10-enanoate (N_2S_2C-11) monolayers on Si;(a) Si 2p; (b) C1s; (c) S 2p; (d) N 1s.
9	**Figure S9.** UPS of 5-(4-phenyl)10,15,20-triphenyl-21,23-dithiaporphyrinyl undec10-enanoate (N_2S_2C-11) monolayers on Si; (a) Work-function calculation from secondary electron cut-offs (WF=4.03 eV); (b) HOMO calculations from WF and BE onset (HOMO=-5.21 eV).
10	**Figure S10.** SIMS-TOF of 5-(4-phenyl)10,15,20-triphenyl-21,23-dithiaporphyrinyl undec10-enanoate (N_2S_2C-11) monolayers on Si.
11	**Figure S11.** Transmission spectrum of Au/ N_2S_2C-11/Au model system calculated by NEGF formulation using ATK software.
12	**Figure S12.** Molecular orbital electron density mapping of N_2S_2C-11 using DFT calculations.
13	**Figure S13.** Overlay of 1000 scans of I-V, for clear representation scans at a gap of 100 scans is shown. (*viz-a-viz* 1st,100th, 200th etc.)

Experimental

Synthesis:

5-(4-Hydroxyphenyl)-10,15,20-tri(phenyl)-21,23-dithiaporphyrin(3).[1] mono-meso-hydroxy 21,23-dithiaporphyrins was synthesized by using Alder's method from unsymmetrical thiophene diols and symmetrical thiatripyrrins. Which were synthesized by literature reported methods.[2,3] (Scheme **1**) A mixture of Diol **1** (1 mmol) and 16-thiatripyrrane **2** (1 mmol) was refluxed in propionic acid (100 mL) for 2 h. The reaction was monitored by UV-Vis spectroscopy and TLC (with a small aliquot, by neutralizing propionic acid. TLC analysis showed the formation of the required porphyrin along with some polymeric material as a base spot. The propionic acid was removed by vacuum distillation and pre-adsorbed in silica gel. The required porphyrin **3** was obtained by silica gel column chromatography with petroleum ether/dichloromethane (15:85), as a purple solid (0.09 g, 8 %). The further purification was done by crystallization in DCM/Hexane. M. P. > 300 ^{o}C, ^{1}H NMR (500 MHz, $CDCl_3$, δ) 9.72 (d, *J* = 5.0 Hz, 1H), 9.68 (d, *J* = 6.5 Hz, 3H), 8.76 – 8.64 (m, 4H), 8.28 – 8.20 (m, 6H), 8.12 (d, *J* = 8.3 Hz, 2H), 7.86 – 7.74 (m, 9H), 7.31 – 7.27 (m, 2H), 5.54 (s, 1H). Elemental analysis: Calcd (%) for $C_{44}H_{28}N_2OS_2$: C, 79.49; H, 4.25; N, 4.21; S, 9.64; Found: C 79.61, H 4.24, N 4.19, S 9.68.[1]

5,10,15,20-tetraphenyl-21,23-dithiaporphyrinyl undec-10-en-oate (N_2S_2 C-11).

To the mixture of Undecenoic acid (0.05 mmol), DCC (0.05 mmol), porphyrin **3** (0.05 mmol) and catalytic amount of 4-dimethylaminopyridine (DMAP) was stirred in CH2Cl2 (15 mL) at room temperature. The progress of reaction was monitored by TLC. After completion of reaction, the byproduct N,N-dicyclohexylurea (DCU) was removed by filtration. The filtrate was washed with water (3 x 10 mL) and dried over anhydrous Na_2SO_4. Solvent was removed under vacuum to give the ester N2S2 C-11, (Scheme 1) which was further purified by passing through a silica gel filtration column using n-hexane–DCM (50:50 v/v) as an eluent. Purple crystals were obtained

after crystallization in DCM/Hexane. M. P. > 300 °C, ^{1}H NMR (400 MHz, $CDCl_3$) δ 9.78 – 9.59 (m, 4H), 8.78 – 8.59 (m, 4H), 8.34 – 8.10 (m, 8H), 7.81 (ddd, J = 4.9, 3.6, 1.5 Hz, 9H), 7.59 – 7.48 (m, 2H), 5.85 (ddt, J = 16.9, 10.2, 6.7 Hz, 1H), 5.10 – 4.90 (m, 2H), 2.75 (t, J = 7.5 Hz, 2H), 2.09 (q, J = 6.9 Hz, 2H), 1.91 (p, J = 7.5 Hz, 2H), 1.89– 1.26 (m, 12H). ^{13}C NMR (100 MHz, $CDCl_3$) δ 172.29, 156.33, 150.81, 147.75, 147.66, 141.04, 139.02, 138.49, 135.37, 135.33, 135.31, 134.81, 134.49, 134.40, 134.01, 127.90, 127.27, 120.49, 114.04, 34.46, 33.65, 29.19, 29.13, 29.04. 28.95, 28.78, 24.91. Elemental analysis: Calcd (%) for $C_{55}H_{46}N_2O_2S_2$: C, 79.49; H, 5.58; N, 3.37; O, 3.85; S, 7.72; Found: C 79.60, H 5.52, N 3.11, S 7.81.

Ccharacterization of monolayers. The monolayers were characterized in terms of thickness, using an ellipsometer (Model: Dimension Icon), surface morphology by AFM imaging (Bruker multimode AFM), de-ionized water contact angle (Holmarc, Contact Angle Meter), FT-IR (Perkin Elmer, Mid IR spectrophotometer, Universal Attenuated Total Reflectance (UATR) with range of TGS and Liquid nitrogen cooled MCT keeping Si-H as the reference and molecular mass by TOF-SIMS in positive polarity (Physical Electronics/PHI TRIFT V NANO TOF) keeping Si-H as the reference. X-ray photoelectron spectroscopy (XPS) using Kratos Axis Ultra X-ray Photoelectron Spectrometer (XPS).

Preparation of H-terminated Si wafers. Highly doped (resistivity: 0.001-0.005 Ωcm) *n*-type silicon (111) wafers were purchased from Chris Baker. The Si (111) wafers were cut into 2x1 cm pieces and were then cleaned by oxidizing the surface with 3:1 (v/v) of conc. H_2SO_4: 30% H_2O_2 (piranha) for 10 min at 80 °C. The wafers were thoroughly washed with H_2O and immersed first in 2% aqueous HF for 2 min and then in 40% de-aerated (purged with N_2 for 30 min) aqueous NH_4F for 10 min to form Hydride terminated Si. The wafers were washed with deionized H_2O and dried under N_2. Hydride formed is stable for 10 minutes but should be immediately transferred to electrochemical.

Monolayer formation. The electro-grafting of **N2S2 C-11** monolayers was done by cyclic voltammetry (CV) using Autolab PGSTAT 30. Where freshly prepared Si-H substrate is used as the working electrode (WE), Pt as the counter electrode (CE) and solid Ag/AgCl as the reference electrode (RE). The solution of 0.1 M tetrabutylammonium perchlorate and **N2S2 C-11** (1μM) in dry CH_2Cl_2 is taken as an electrolyte. The CV was performed under the negative potential of 0 to -1 V at a scan rate of 0.05 V/s under an inert atmosphere for 25 scans, monitored by disappearance of reduction peak of terminal alkenyl bond of **N2S2 C-11**.

Junction and measurement setup. The I–V curves were recorded using a Keithley 6400 using mercury drop as soft top contact.

Theoretical calculations. The ground state geometry was optimized by DFT calculation using ab-initio LCAO-MO approach in Gaussian software and electron density maps of orbitals were calculated. The electron-transport across the Au/N2S2 C-11/Au junctions was investigated by the ATK 11.2.3 program, using a semi-empirical extended Hückel theory and the first-principle NEGF.

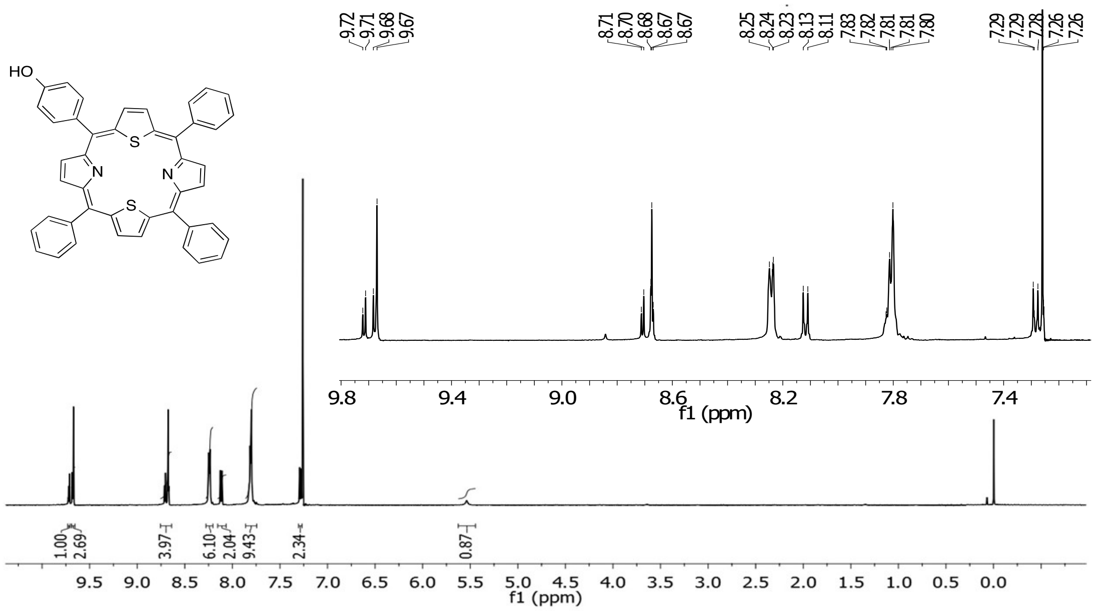

Figure S1. ^{1}H NMR of 5-(4-hydroxy phenyl)10,15,20-triphenyl-21,23-dithiaporphyrin

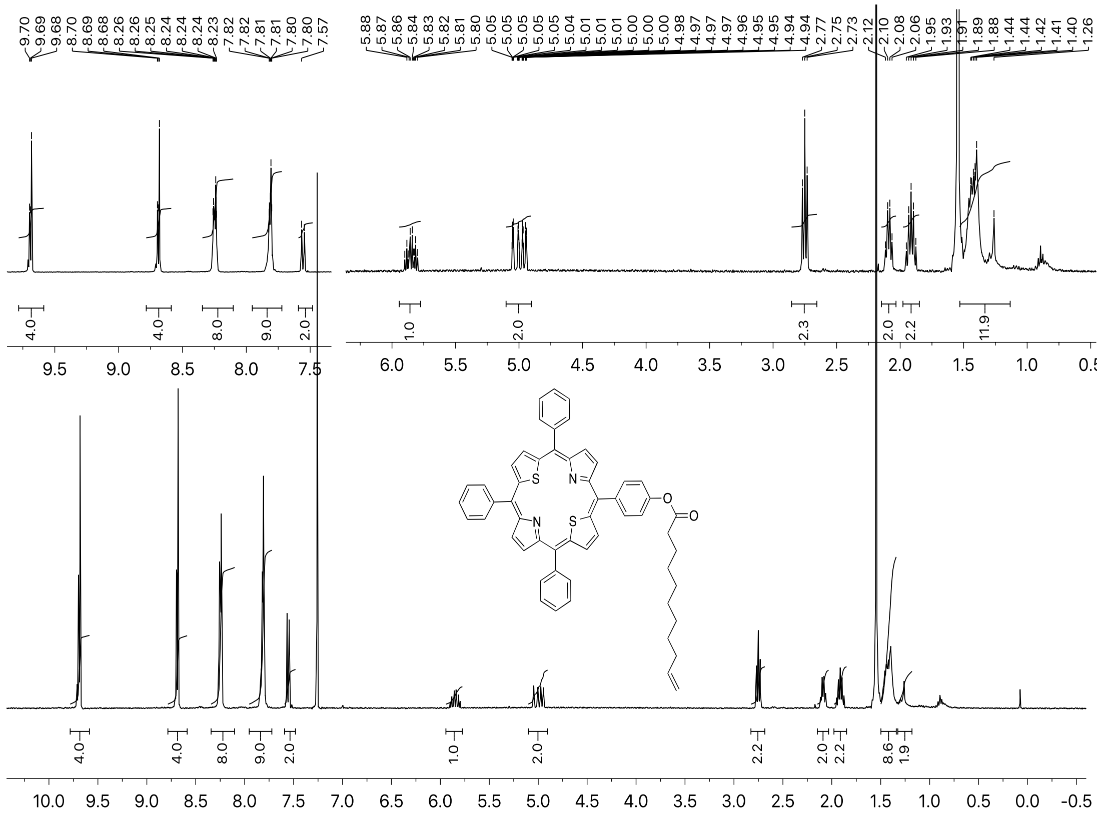

Figure S2. ^{1}H NMR of 5-(4-phenyl)10,15,20-triphenyl-21,23-dithiaporphyrinyl undec10-enanoate (N_2S_2C-11).

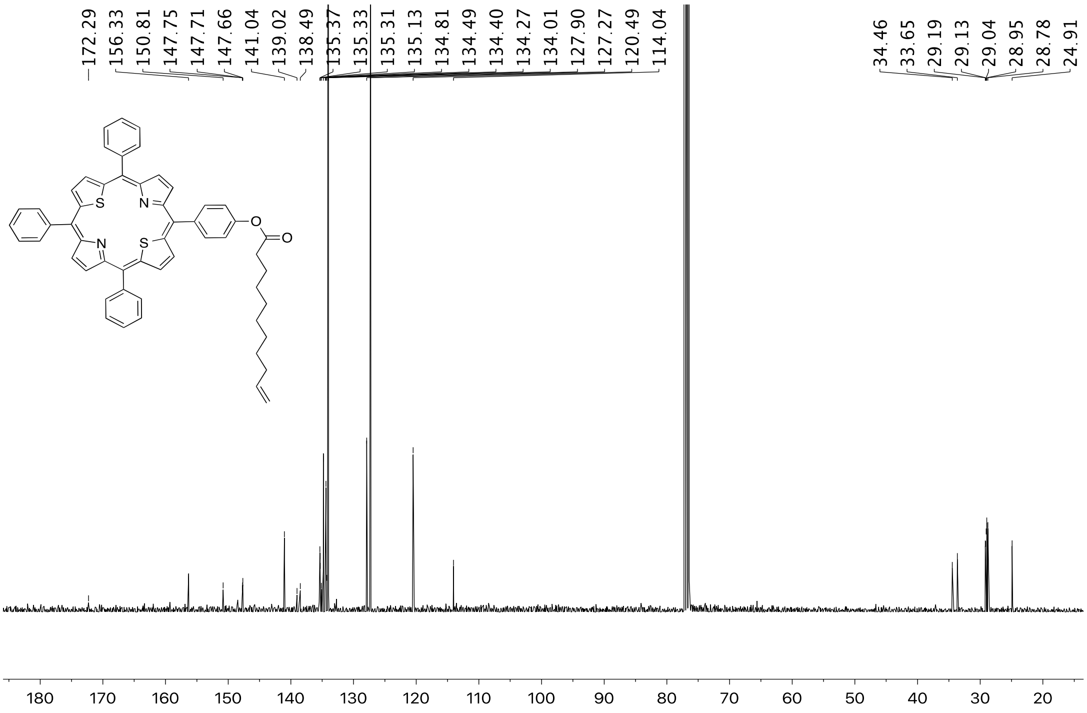

Figure S3. ^{13}C NMR of 5-(4-phenyl)10,15,20-triphenyl-21,23-dithiaporphyrinyl undec10-enanoate (N_2S_2C-11).

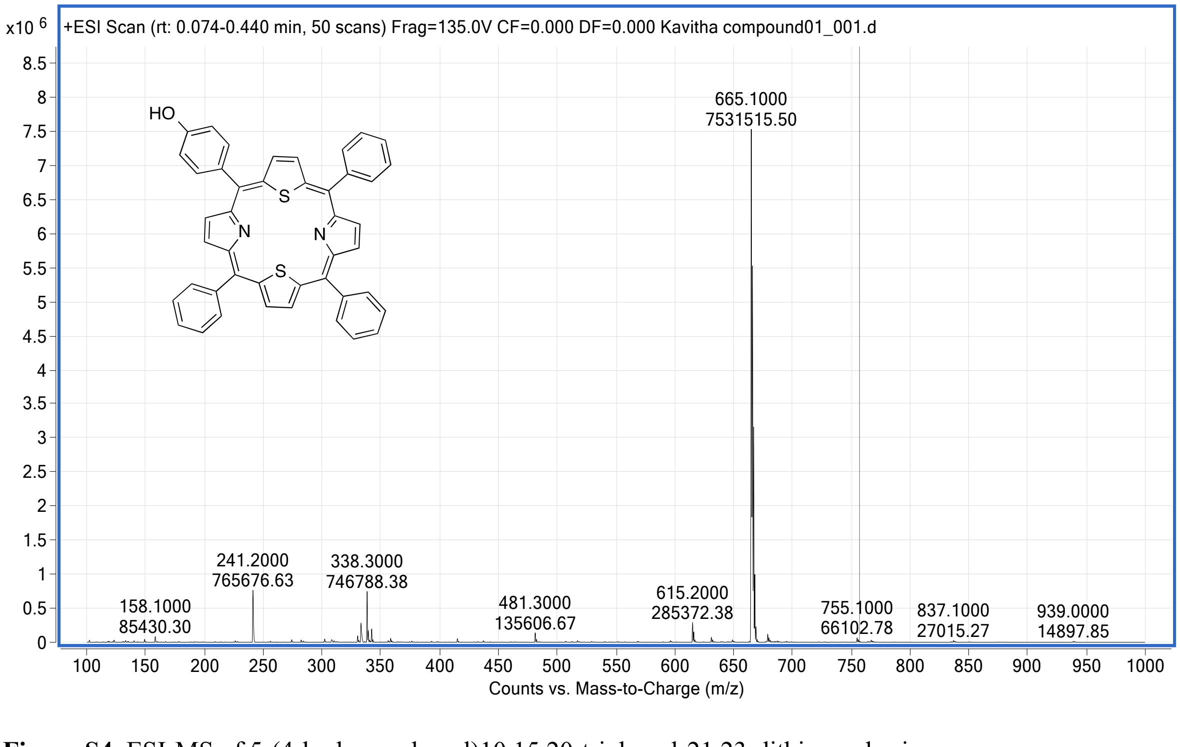

Figure S4. ESI-MS of 5-(4-hydroxy phenyl)10,15,20-triphenyl-21,23-dithiaporphyrin

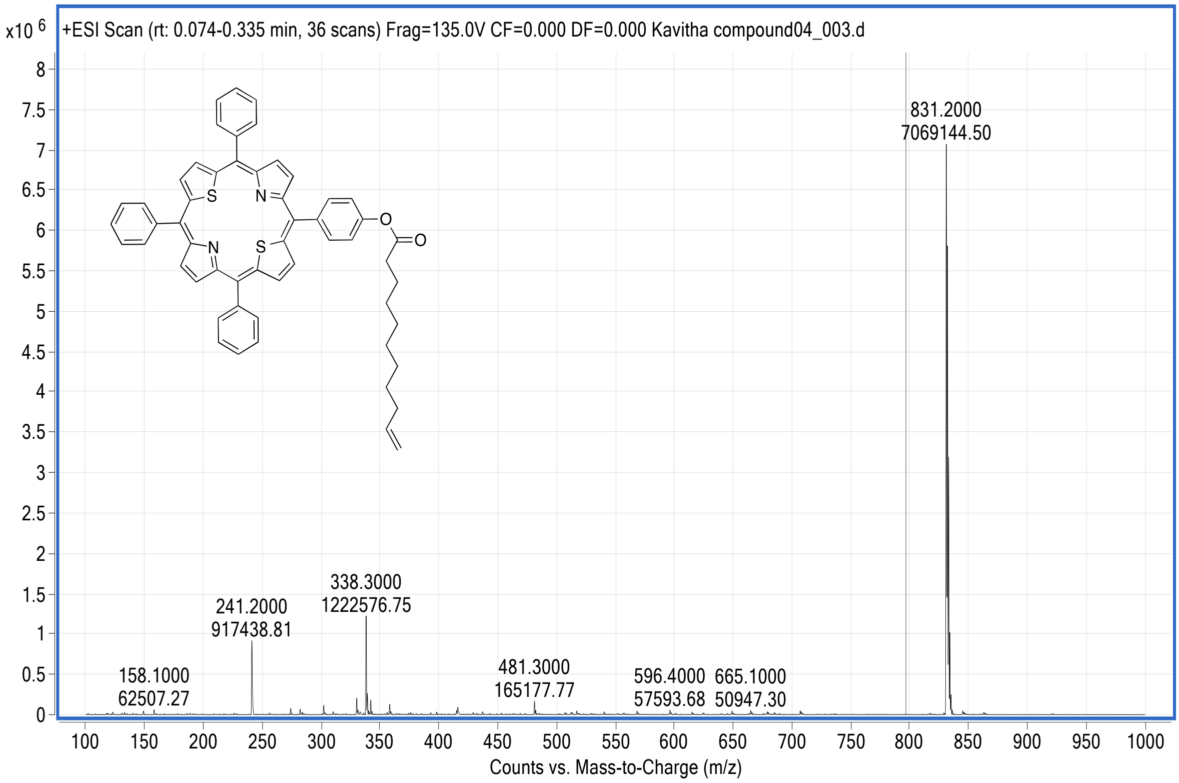

Figure S5. ESI-MS of 5-(4-phenyl)10,15,20-triphenyl-21,23-dithiaporphyrinyl undec10-enanoate (N_2S_2C-11)

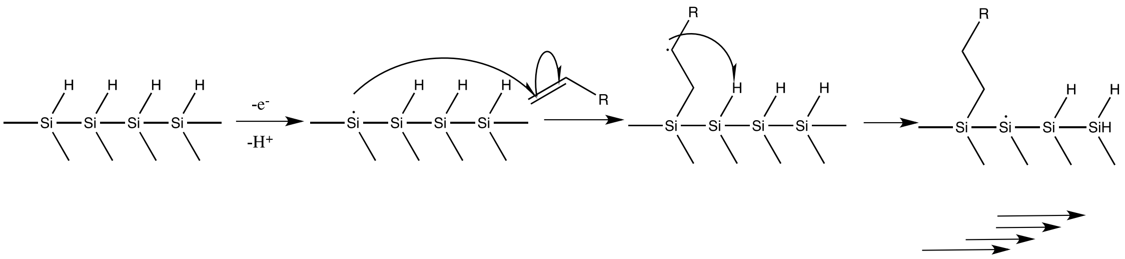

Figure S6. Mechanism of monolayer formation

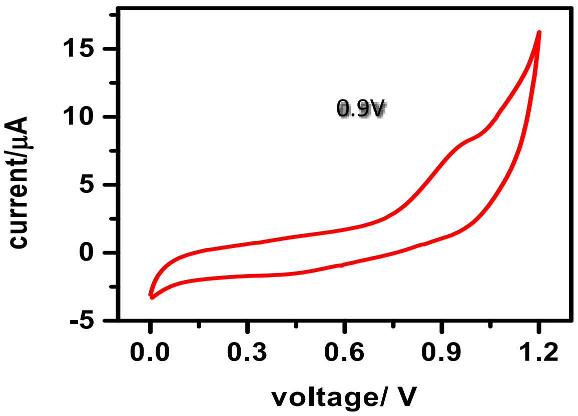

Figure S7. CV of N2S2 solution

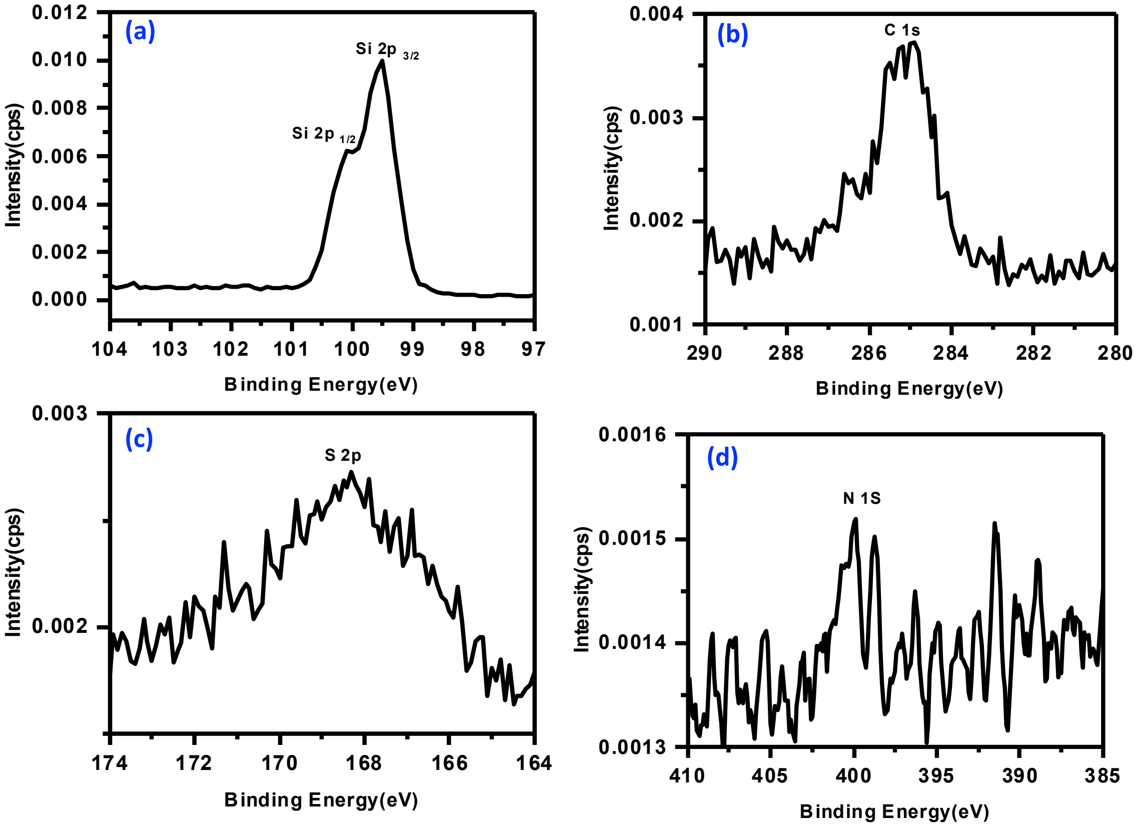

Figure S8. XPS of 5-(4-phenyl)10,15,20-triphenyl-21,23-dithiaporphyrinyl undec10-enanoate (N_2S_2C-11) monolayers on Si;(a) Si 2p; (b) C1s; (c) S 2p; (d) N 1s.

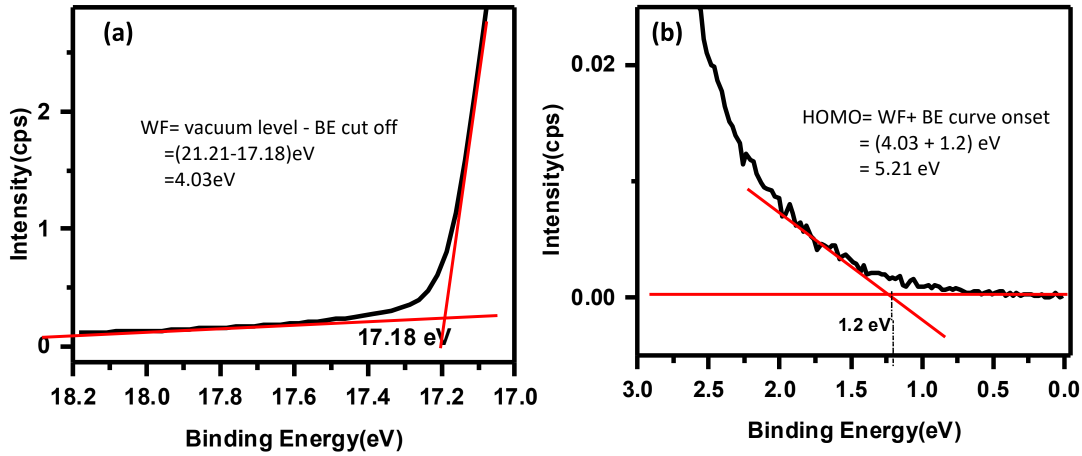

Figure S9. UPS of 5-(4-phenyl)10,15,20-triphenyl-21,23-dithiaporphyrinyl undec10-enanoate (N_2S_2C-11) monolayers on Si; (a) Work-function calculation from secondary electron cut-offs (WF=4.03 eV); (b) HOMO

calculations from WF and BE onset (HOMO=-5.21 eV SIMS-TOF of 5-(4-phenyl)10,15,20-triphenyl-21,23-dithiaporphyrinyl undec10-enanoate (N_2S_2C-11) monolayers on Si

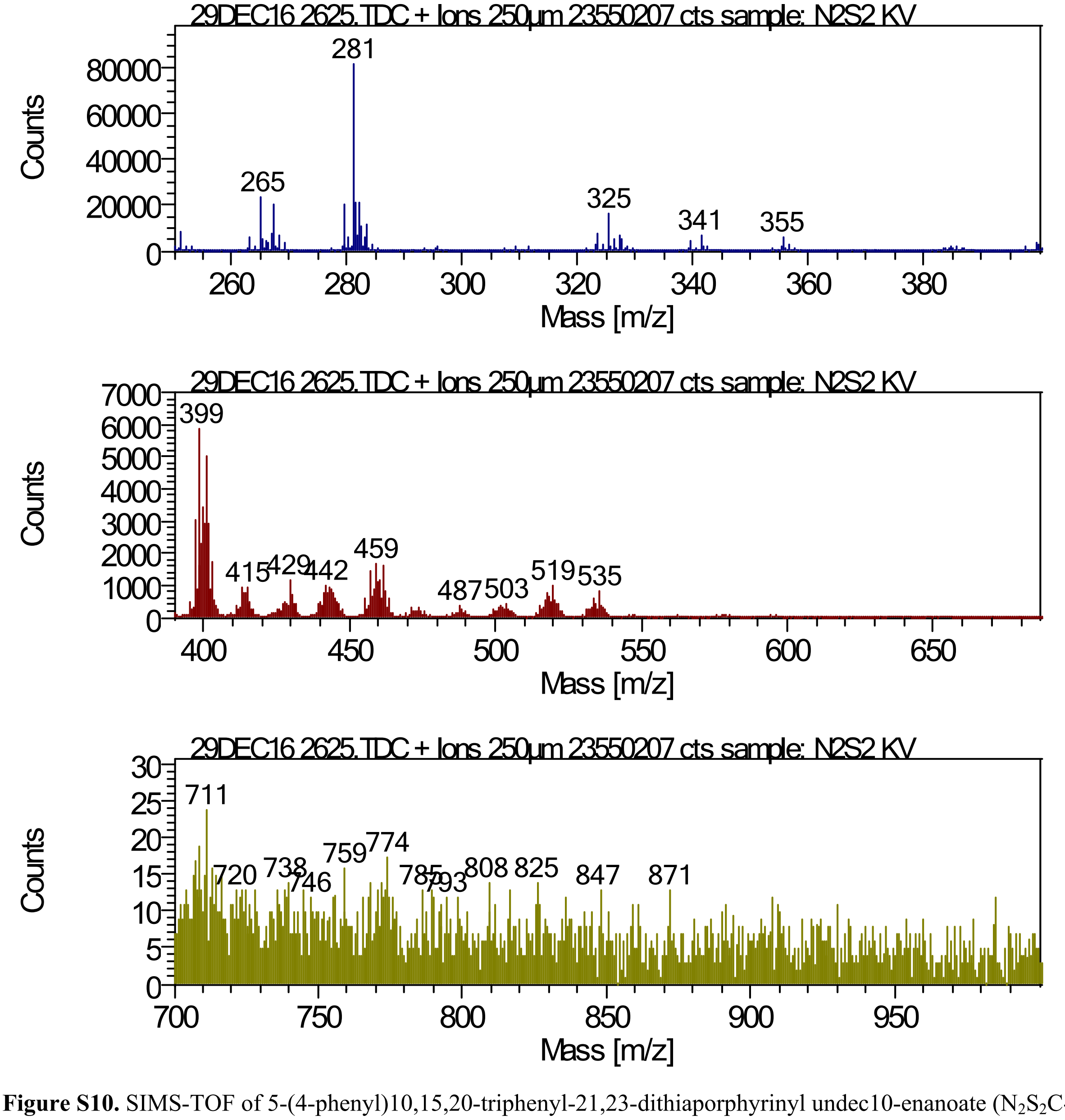

Figure S10. SIMS-TOF of 5-(4-phenyl)10,15,20-triphenyl-21,23-dithiaporphyrinyl undec10-enanoate (N_2S_2C-11) monolayers on Si.

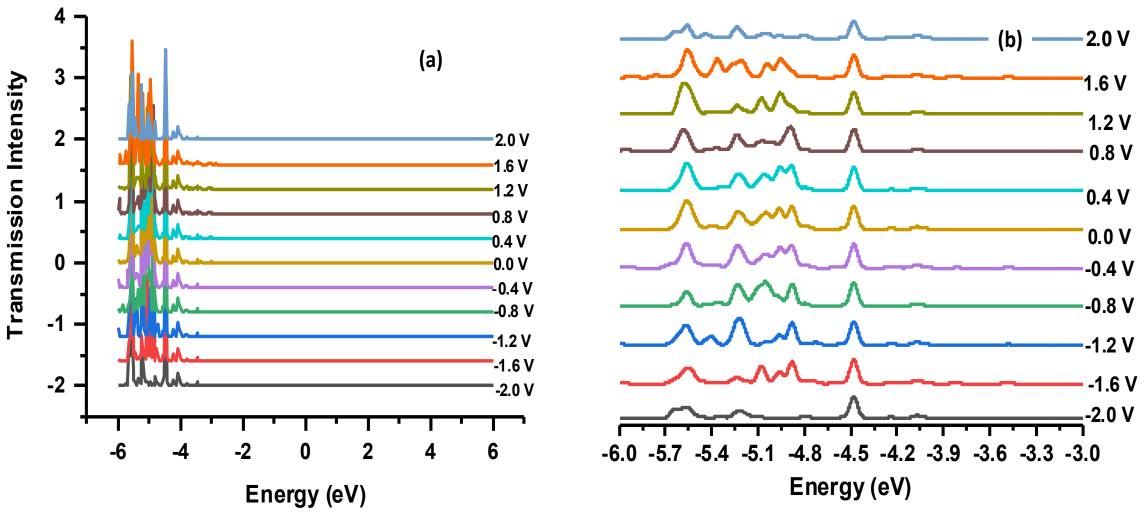

Figure S11. Transmission spectrum of Au/ N_2S_2C-11/Au model system calculated by NEGF formulation using ATK software at different applied Bias (a) Full spectrum (b) Expansion.

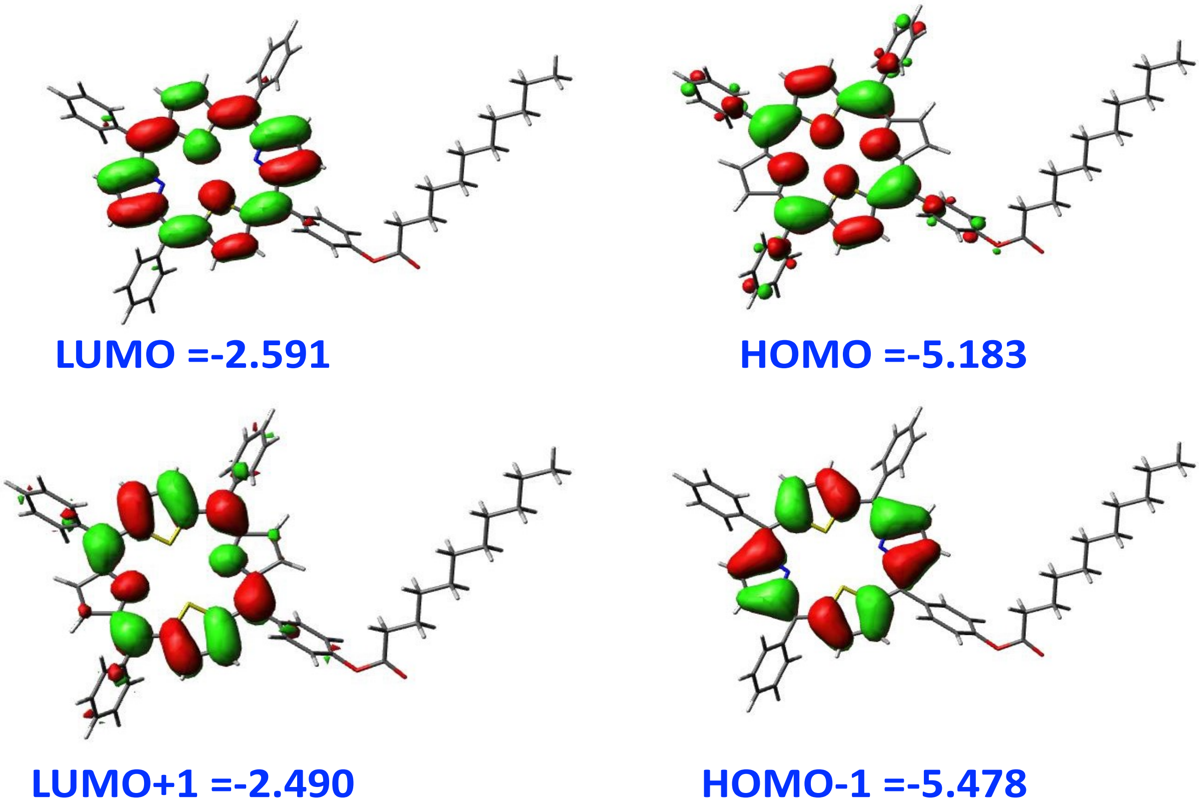

Figure S12. Molecular orbital electron density mapping of N_2S_2C-11 using DFT calculations.

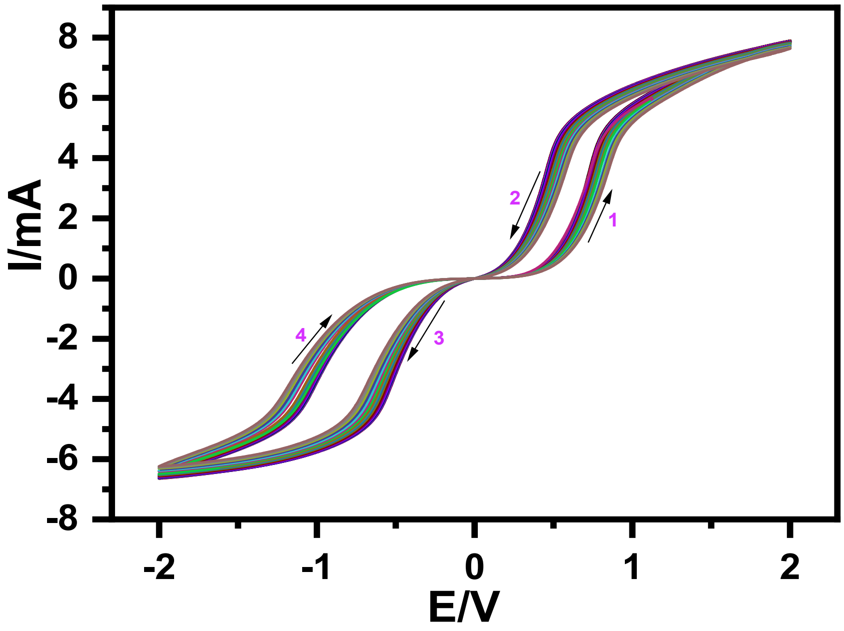

Figure S13. Overlay of 1000 scans of I-V, for clear representation scans at a gap of 100 scans is shown. (*viz-a-viz* 1st,100th, 200th etc.)

References

1 K. Garg, R. Shanmugam and P. C. Ramamurthy, *Carbon N. Y.*, 2017, **122**, 307–318.

2 A. D. Bromby, W. H. Kan and T. C. Sutherland, *J. Mater. Chem.*, 2012, **22**, 20611.

3 S. Punidha, N. Agarwal, R. Burai and M. Ravikanth, *European J. Org. Chem.*, 2004, 2223–2230.
